# A novel long noncoding RNA HOXC-AS3 mediates tumorigenesis of gastric cancer by binding to YBX1

**DOI:** 10.1186/s13059-018-1523-0

**Published:** 2018-10-04

**Authors:** Erbao Zhang, Xuezhi He, Chongguo Zhang, Jun Su, Xiyi Lu, Xinxin Si, Jinfei Chen, Dandan Yin, Liang Han, Wei De

**Affiliations:** 1 Department of Epidemiology and Biostatistics, Jiangsu Key Lab of Cancer Biomarkers, Prevention and Treatment, Collaborative Innovation Center for Cancer Personalized Medicine, School of Public Health, Nanjing Medical University, Nanjing, Jiangsu People’s Republic of China; 2 Department of Biochemistry and Molecular Biology, Nanjing Medical University, Nanjing, Jiangsu People’s Republic of China; 3 Department of Oncology, Second Affiliated Hospital of Nanjing Medical University, Nanjing, Jiangsu People’s Republic of China; 4 Department of Oncology, The Affiliated Jiangyin Hospital of Southeast University Medical College, Jiangyin, Jiangsu People’s Republic of China; 5 Department of Oncology, First Affiliated Hospital of Nanjing Medical University, Nanjing, Jiangsu People’s Republic of China; 6 Huaihai Institute of Technology, Lianyungang, Jiangsu People’s Republic of China; 7 Department of Oncology, Nanjing First Hospital, Nanjing Medical University, Nanjing, Jiangsu People’s Republic of China; 8 Cancer Research and Biotherapy Center, Nanjing Second Hospital, the Second Affiliated Hospital of Southeast University, Nanjing, Jiangsu People’s Republic of China; 9 Department of Oncology, Xuzhou Central Hospital, Affiliated Xuzhou Hospital, College of Medicine, Southeast University, Xuzhou, Jiangsu People’s Republic of China; 10 Xuzhou Clinical School of Xuzhou Medical University, Xuzhou, Jiangsu People’s Republic of China

**Keywords:** Histone modification, HOXC-AS3, YBX1, GC

## Abstract

**Background:**

Recently, increasing evidence shows that long noncoding RNAs (lncRNAs) play a significant role in human tumorigenesis. However, the function of lncRNAs in human gastric cancer remains largely unknown.

**Results:**

By using publicly available expression profiling data from gastric cancer and integrating bioinformatics analyses, we screen and identify a novel lncRNA, HOXC-AS3. HOXC-AS3 is significantly increased in gastric cancer tissues and is correlated with clinical outcomes of gastric cancer. In addition, HOXC-AS3 regulates cell proliferation and migration both in vitro and in vivo. RNA-seq analysis reveals that HOXC-AS3 knockdown preferentially affects genes that are linked to proliferation and migration. Mechanistically, we find that HOXC-AS3 is obviously activated by gain of H3K4me3 and H3K27ac, both in cells and in tissues. RNA pull-down mass spectrometry analysis identifies that YBX1 interacts with HOXC-AS3, and RNA-seq analysis finds a marked overlap in genes differentially expressed after YBX1 knockdown and those transcriptionally regulated by HOXC-AS3, suggesting that YBX1 participates in HOXC-AS3-mediated gene transcriptional regulation in the tumorigenesis of gastric cancer.

**Conclusions:**

Together, our data demonstrate that abnormal histone modification-activated HOXC-AS3 may play important roles in gastric cancer oncogenesis and may serve as a target for gastric cancer diagnosis and therapy.

**Electronic supplementary material:**

The online version of this article (10.1186/s13059-018-1523-0) contains supplementary material, which is available to authorized users.

## Background

Gastric cancer is one of the leading causes of cancer-related deaths worldwide and the most common gastrointestinal malignancy in East Asia [1, 2]. Gastric cancer is diagnosed at an advanced stage accompanied by malignant proliferation in most patients, and the prognosis for advanced stage patients remains very poor [3]. Therefore, additional research is needed to discover and develop effective biomarkers and targets for gastric cancer diagnosis and treatment.

To date, gastric cancer research has mainly focused on the deregulation of protein-coding genes to identify oncogenes and tumor suppressors that could serve as diagnostic and therapeutic targets. However, protein-coding sequences occupy less than 2% of the human genome [4, 5]. LncRNAs are operationally defined as RNA transcripts that are > 200 nt with limited protein coding potential [6], which have been shown to play a key role in tumorigenesis, including GC [7–9]. Many studies found that lncRNAs could play an important role in regulating gene expression by different mechanisms, including chromatin modification, and transcriptional and posttranscriptional processing [10–12]. For example, HOTAIR is involved in the transcriptional repression of HOX loci and promotes breast metastasis by binding to PRC2 (Polycomb Repressive Complex) [13]. However, the biological functions of lncRNAs in the control of GC tumorigenesis are not well characterized. Therefore, a better understanding of the role of lncRNAs underlying GC progression will enrich the understanding of the molecular mechanisms of GC carcinogenesis and provide information for improving the diagnosis and treatment of GC.

In our present study, we identified the full sequence of HOXC-AS3 and found that gain of H3K4me3 and H3K27acetylation could activate the expression of HOXC-AS3, both in cells and in tissues. HOXC-AS3 was also significantly upregulated in GC tissues compared with the corresponding nontumor tissues and may serve as an independent predictor for the overall survival in GC. In addition, HOXC-AS3 regulated cell proliferation and migration both in vitro and in vivo. RNA-seq analysis for whole transcriptome studies indicates an important role for HOXC-AS3 in the tumorigenesis of GC, and the activated function of HOXC-AS3 was mediated, in part, by interaction with YBX1. These results suggest that further studies to identify nonprotein-coding genes that contribute to oncogenesis are necessary for elucidating the complex genetic rewiring that is driven by HOXC-AS3 in GC.

## Results

### Identification of HOXC-AS3 by analyzing gastric cancer RNA-expression profiling data

**Fig. 1:**
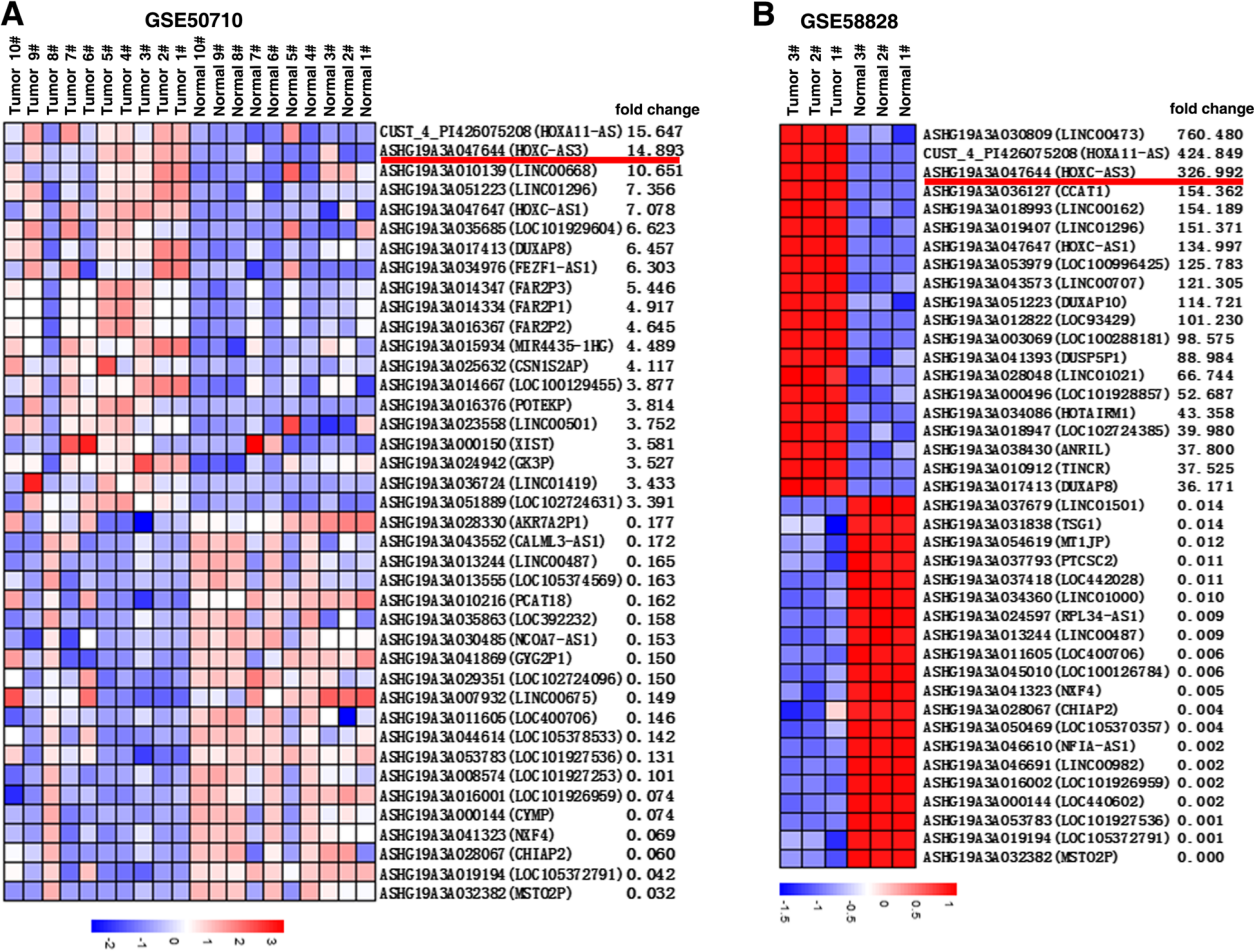
Screening HOXC-AS3 by bioinformatics analysis. **a**, **b** Raw microarray data were downloaded from GEO, including GSE50710 (*n* = 20) and GSE58828 (*n* = 6). Then, the raw data were normalized and z-score-transformed using RMAExpress. The top 20 dysregulated lncRNAs in these GEO datasets are shown in **a** and **b**

**Fig. 2:**
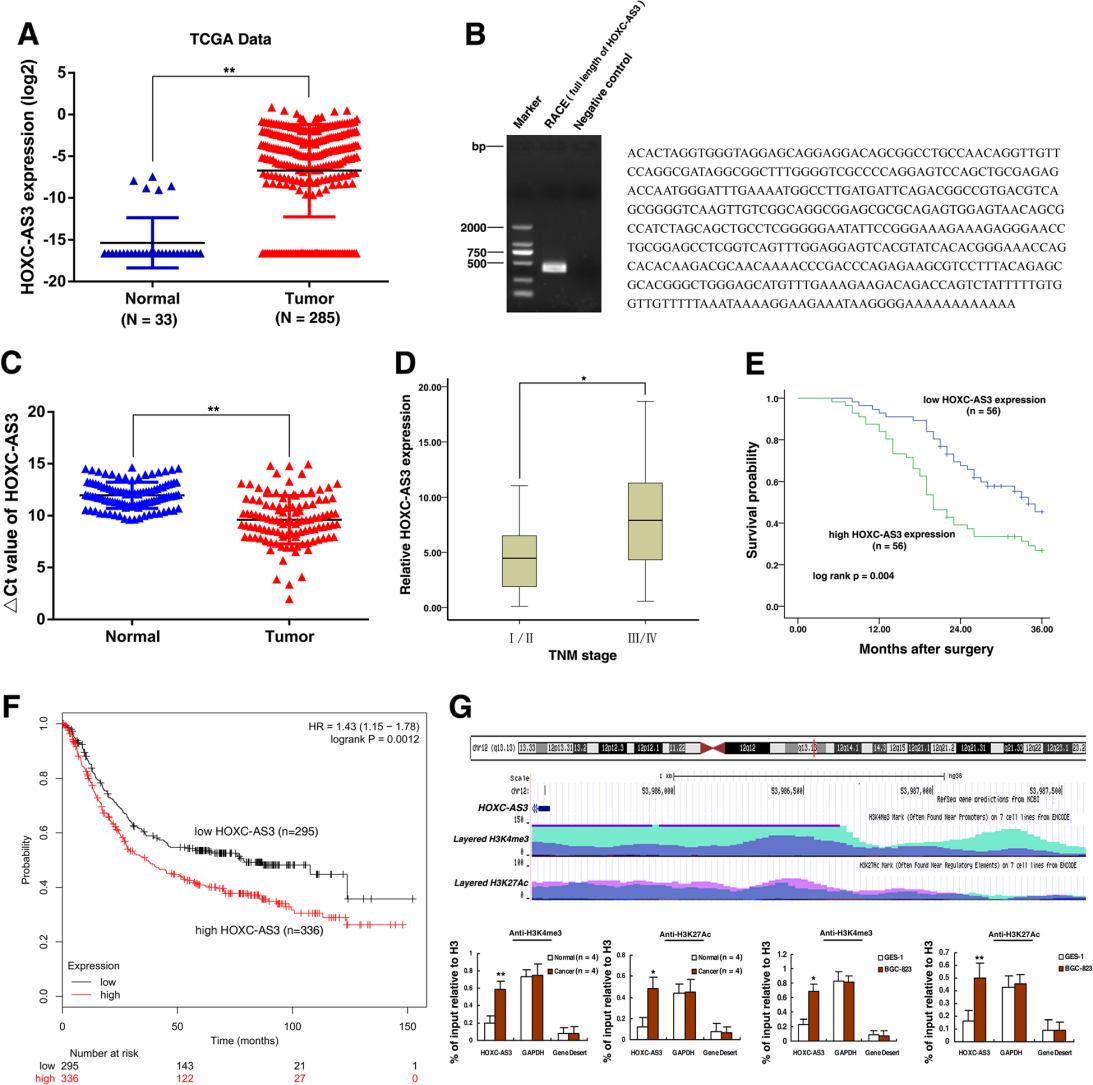
Identification of HOXC-AS3 and expression of HOXC-AS3 in GC tissues and its clinical parameters and gain of H3K4me3 and H3K27 acetylation could activate HOXC-AS3 in GC. **a** HOXC-AS3 expression in GC tissues (*n* = 285) compared with noncancerous tissues (*n* = 33) analyzed using the TCGA database (data from TANRIC). **b** The full sequence of HOXC-AS3 was confirmed by RACE. **c** HOXC-AS3 was detected in 112 pairs of GC tissues by qRT-PCR. The levels of HOXC-AS3 in GC tissues are significantly higher than in nontumor tissues. The ΔCt value was determined by subtracting the GAPDH Ct value from the HOXC-AS3 Ct value. A smaller ΔCt value indicates higher expression. **d** HOXC-AS3 expression was significantly higher in patients with an advanced TNM stage. **e** Patients with high levels of HOXC-AS3 expression showed reduced survival times compared with patients with low levels of HOXC-AS3 expression (*P* < 0.01, log-rank test). **f** Kaplan–Meier survival plots demonstrating that high HOXC-AS3 expression levels correlated with worse OS in GC patients (*n* = 631). **g** The UCSC Genome Bioinformatics Site (http://genome.ucsc.edu/) showed high enrichment of H3K4me3 and H3K27Ac at the promoter of HOXC-AS3. ChIP assays detected the level of H3K4me3 and H3K27Ac at the promoter of HOXC-AS3 in GC tissues and cells. *P < 0.05, **P < 0.01

Raw microarray data was downloaded from GEO, including GSE50710 (*n* = 20) and GSE58828 (*n* = 6). To obtain differentially expressed lncRNAs, signal data were normalized and z-score-transformed (paired or group *t*-test according to the experimental design was used to validate statistical significance). The top 20 dysregulated lncRNAs in these GEO datasets were shown in Fig. 1a, b. For example, the lncRNA that showed clear upregulation was HOXA11-AS (the largest increase in GSE50710), and our previous study showed that HOXA11-AS could regulate cell proliferation and invasion of gastric cancer by scaffolding chromatin modification factors and patients with high HOXA11-AS expression had a shorter survival and poorer prognosis [14]. The most obvious increase in GSE58828, LINC00473, could mediate tumor growth and elevated LINC00473 expression correlated with poor prognosis of lung cancer [15]. Our previous study showed that FEZF1-AS1 could promote gastric cancer proliferation and higher expression of FEZF1-AS1 predicted poor prognosis [16]. Qin et al. found that LINC01296 was upregulated in GC tissue and correlated with poor prognosis [17]. CCAT1 is an oncogenic long noncoding RNA in human cancers including GC [18]. Our previous work found that CCAT1 could regulate cell proliferation and migration in esophageal squamous cell carcinoma and higher expression of CCAT1 is correlated with poorer outcomes [19]. In addition, CCAT1 could serve as important prognostic biomarkers in colorectal cancer [20]. We focused on overexpressed lncRNAs because these lncRNAs may be more readily used as early diagnosis markers or therapeutic targets. Therefore, although a small portion of lncRNAs have been functionally characterized, many members in the class remain uncharacterized. In our analysis of the results, HOXC-AS3 was the lncRNA clearly upregulated in both GSE50710 and GSE58828 (the rank of HOXC-AS3 is in the exceeding front position, both in GSE50710 and GSE58828), and the role in GC tumorigenesis including other cancer types remained unclear. This prompted us to explore the role of HOXC-AS3 in human GC. To further validate this result, we analyzed that RNA-Seq data (from The Cancer Genome Atlas (TCGA)) of lncRNAs of gastric cancer were from TANRIC [21] (http://ibl.mdanderson.org/tanric/_design/basic/index.html). As shown in Fig. 2a, HOXC-AS3 was significantly upregulated in GC tissues from the TCGA data.

### Gain of H3K4me3 and H3K27 acetylation-activated HOXC-AS3 is upregulated in human GC tissues and correlates with poor prognosis

For further study, we first performed rapid amplification of cDNA ends (RACE) to identify the full sequence of HOXC-AS3 in BGC-823 cells according the sequence archived in the RefSeq database of NCBI (440 bp, with poly (A) tail, Fig. 2b). To validate the expression results from high-throughput data, as shown in Fig. 2c, the HOXC-AS3 expression level in tumor tissues was significantly higher in 112 pairs of GC tumor tissues compared with adjacent normal tissues (*P* < 0.001). One tumor tissue showed an upregulation of HOXC-AS3 greater than 521-fold relative to normal tissue. Next, we explored the correlation between HOXC-AS3 expression and the clinicopathological factors of patients with GC. The result showed that the HOXC-AS3 level was associated with TNM stage. Patients with advanced TNM stage (III/IV) were associated with higher HOXC-AS3 expression, whereas patients with local TNM stage (I/II) were associated with a lower HOXC-AS3 level (27.1317 ± 80.26254 vs 4.7483 ± 3.02402, *P* = 0.046) (Fig. 2d). Furthermore, we divided the samples into relatively high (above the mean, *n* = 56) and relatively low (below the mean, *n* = 56) HOXC-AS3 expression groups according to the median value of HOXC-AS3 levels. A chi-square test was then performed to evaluate clinicopathological factors between the two groups. As shown in Additional file 1: Table S1, the relative HOXC-AS3 level was also correlated with histological grade (*P* = 0.002), tumor invasion depth (*P* = 0.008), lymph node metastasis (*P* = 0.035), and TNM stage (*P* = 0.002). No relationship between HOXC-AS3 expression and other clinical factors, such as sex (male, female) and patient age (≤ 60, > 60), was found in our study.

To determine the relationship between HOXC-AS3 expression and GC patient prognosis, we evaluated the correlation between HOXC-AS3 expression and clinical outcomes. Kaplan–Meier analysis and log-rank test were used to evaluate the effects of HOXC-AS3 expression and the clinicopathological characteristics on overall survival (OS). The median survival time for low HOXC-AS3 expression groups was 34 months, whereas for high HOXC-AS3 expression groups, it was only 20 ± 1.357 months. As shown in Fig. 2e, overexpression of HOXC-AS3 predicted a poor prognosis in patients with GC (*P* = 0.004). Similarly, the correlation between HOXC-AS3 expression levels and the survival of GC patients was also supported by Kaplan–Meier Plotter analysis (http://kmplot.com/analysis/, detailed steps were described in Additional file 8: Supplementary Methods), which indicated that higher HOXC-AS3 expression correlated with worse OS, using publicly available chip data from 631 GC patients (Fig. 2f).

Then, univariate and multivariate survival analyses (Cox proportional hazards regression model) were performed. Univariate analysis identified two prognostic factors: TNM stage and HOXC-AS3 expression. Multivariate analysis further revealed that HOXC-AS3 expression was an independent predictor for overall survival in patients with GC (*P* < 0.001), as well as TNM stage (*P* = 0.023) (Additional file 2: Table S2).

To explore the mechanism of high expression of HOXC-AS3 in GC, firstly, by using the UCSC Genome Bioinformatics Site (http://genome.ucsc.edu/), we found high enrichment and overlaps of H3K4me3 and H3K27Ac peaks at the promoter region of HOXC-AS3 (Fig. 2g, H3K4me3 and H3K27Ac, two markers of active promoters). Using ChIP assays, we found gain of H3K4me3 and H3K27Ac in cancer tissues compared with normal tissues (*n* = 4) at the promoter of HOXC-AS3. We also observed the gain of H3K4me3 and H3K27Ac in GC cells (BGC-823) compared with normal human esophageal epithelial cells (GES-1) at the promoter of HOXC-AS3 (Fig. 2g). Taken together, these data confirm that HOXC-AS3 is frequently increased in GC. Abnormal histone modification, gain of histone sites H3K4me3 and H3K27Ac of the promoter may partially account for the significant activation of HOXC-AS3.

### HOXC-AS3 regulates GC cell proliferation and migration in vitro

**Fig. 3:**
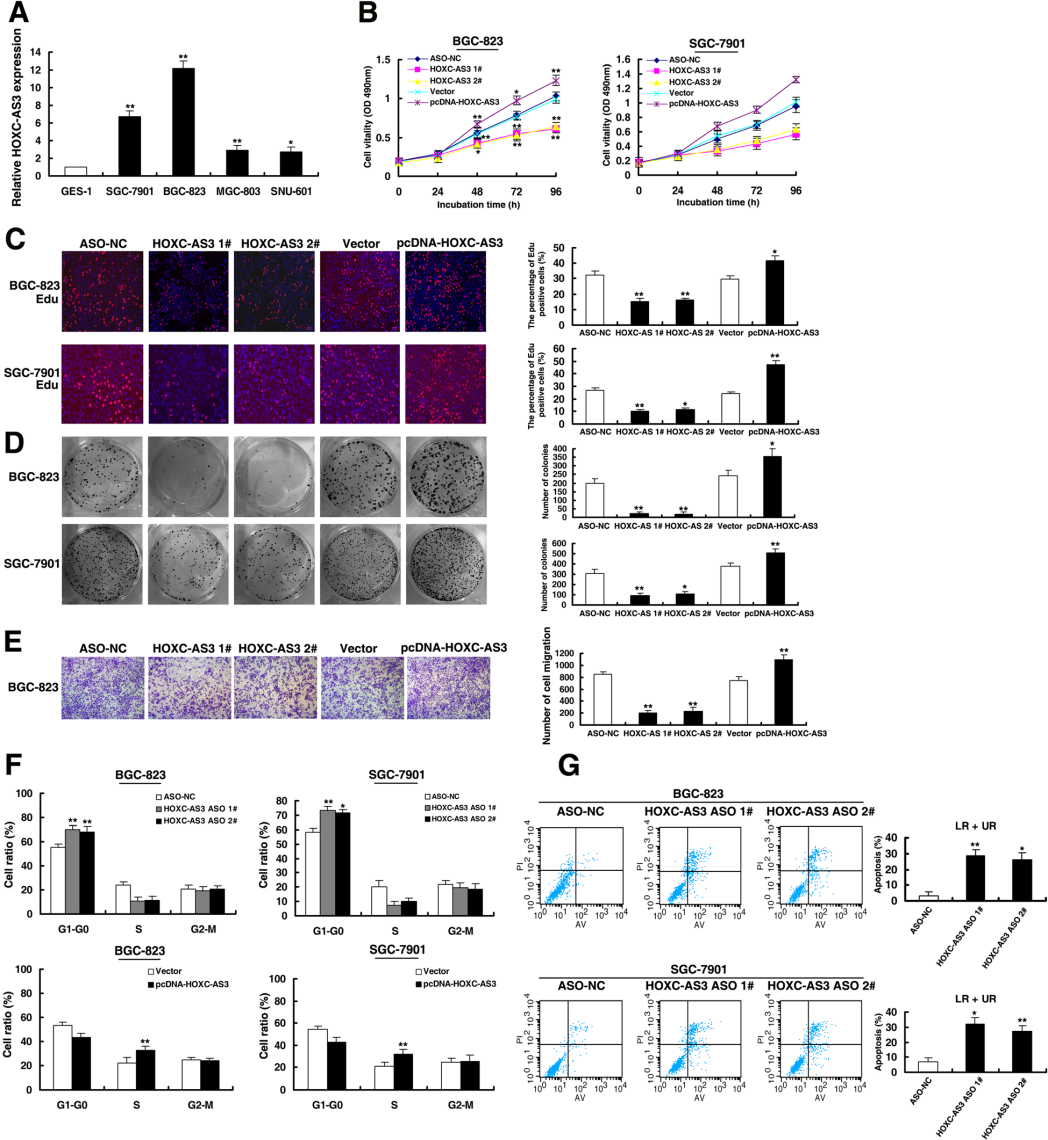
HOXC-AS3 regulates GC cell proliferation and migration in vitro. **a** Analysis of HOXC-AS3 expression levels in GC cell lines (SGC-7901, BGC-823, MGC-803, and SNU-601) compared with a normal gastric epithelium cell line (GES-1) by qRT-PCR. **b** MTT assays were performed to determine cell proliferation of BGC-823 and SGC-7901 cells after transfection of overexpression plasmid and ASO of HOXC-AS3. **c** EdU assays of BGC-823 and SGC-7901 cells transfected with an overexpression plasmid and knockdown of HOXC-AS3. **d** Colony formation assays of BGC-823 and SGC-7901 cells transfected with an overexpression plasmid and knockdown of HOXC-AS3. **e** Transwell assays were used to investigate the changes in migratory abilities of BGC-823 cells after transfection, respectively. **f** At 48 h after transfection, cell cycle was analyzed by flow cytometry. The bar chart represents the percentage of cells in G1–G0, S, or G2–M phase, as indicated. **g** At 48 h after transfection, BGC-823 and SGC-7901 cells were stained and analyzed by flow cytometry. LR, early apoptotic cells. UR, terminal apoptotic cells. *P < 0.05, **P < 0.01

To explore the role of HOXC-AS3 in GC, we examined the HOXC-AS3 expression levels in gastric cancer cell lines. As shown in Fig. 3a, gastric cancer cell lines expressed significantly higher levels of HOXC-AS3 than a normal gastric epithelium cell line (GES-1). To further confirm the function of HOXC-AS3, we used an LNA-ASO (Locked Nucleic Acid, antisense oligonucleotide) targeting HOXC-AS3 and control LNA-ASO. As shown in (Additional file 3: Figure S1A), the ASO-mediated knockdown and plasmid-mediated overexpression were used for exogenously manipulating the expression of HOXC-AS3 in BGC-823 and SGC-7901 cells. Then, MTT assays showed that knockdown of HOXC-AS3 expression significantly inhibited cell proliferation compared with control cells. By contrast, overexpressed HOXC-AS3 promoted cell proliferation (Fig. 3b). EdU assays demonstrated that HOXC-AS3 had a significant impact on GC cell proliferation (Fig. 3c). Similarly, the results of the colony-formation assay revealed that clonogenic survival was significantly decreased following knockdown of HOXC-AS3. Furthermore, overexpression of HOXC-AS3 could increase the number of clones (Fig. 3d). Next, transwell assays revealed that knockdown of HOXC-AS3 significantly repressed cell migration compared with the control in BGC-823 cells. By contrast, overexpression of HOXC-AS3 promoted cell migration (Fig. 3e). Flow cytometric analysis was performed to further examine whether HOXC-AS3 affected the proliferation of GC cells by altering cell-cycle progression. The results revealed that cell-cycle progression of knockdown-HOXC-AS3 cells was significantly stalled at the G1-G0 phase compared with controls both in BGC-823 and in SGC-7901 cells. Overexpressed HOXC-AS3 promoted S phase progression (Fig. 3f). Additionally, knockdown of HOXC-AS3 significantly induced BGC-823 and SGC-7901 cell apoptosis (Fig. 3g).

### HOXC-AS3 regulates GC cell proliferation and migration in vivo

**Fig. 4:**
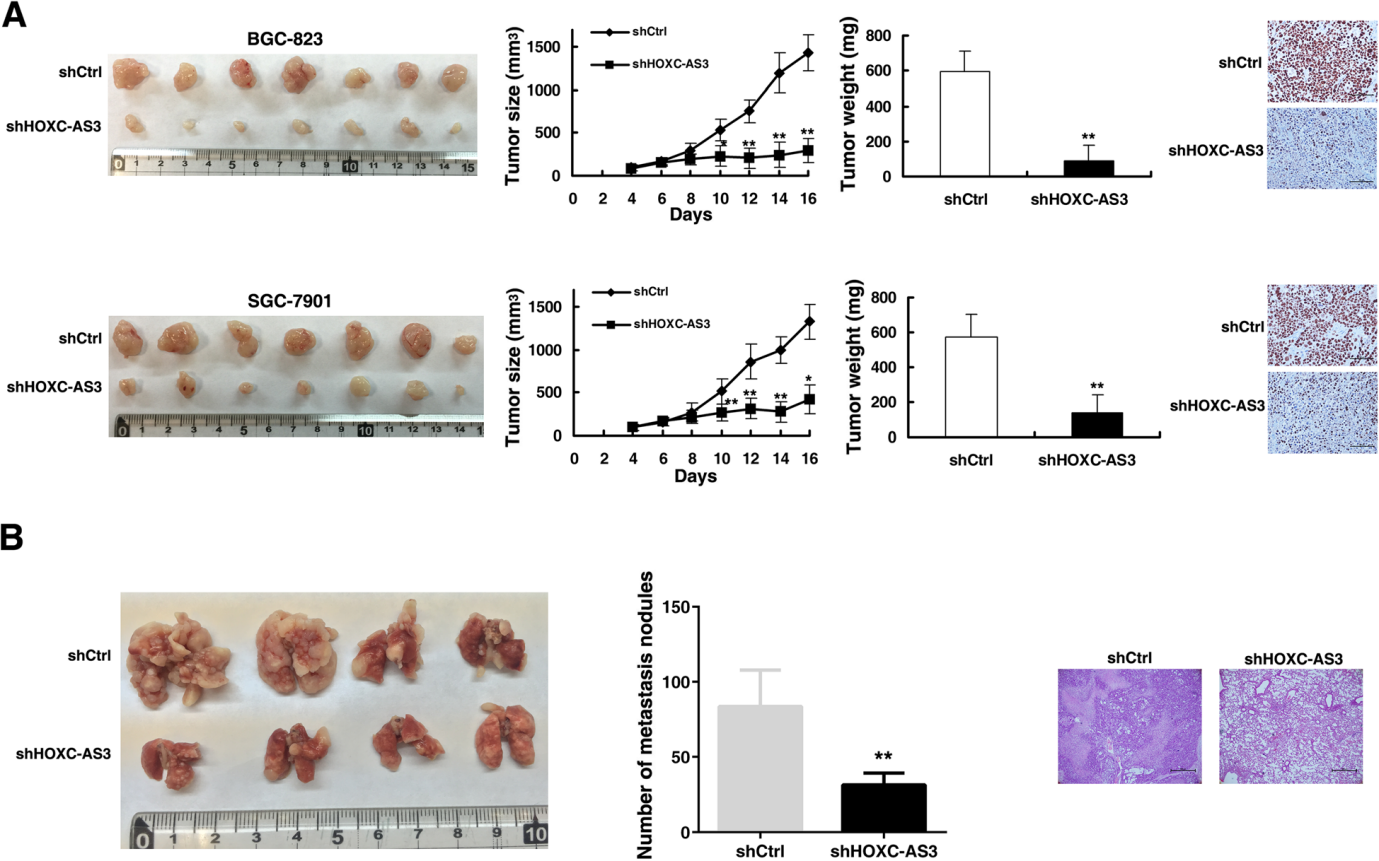
HOXC-AS3 regulates GC cell proliferation and migration in vivo*.* **a** Scramble or shHOXC-AS3 was stably transfected into BGC-823 and SGC-7901 cells, which were injected into nude mice, respectively. Tumor volumes were calculated after injection every 2 days. Tumor weights are represented as the means of tumor weights ± S.D. (standard deviation). The qRT-PCR was performed to detect the average expression of HOXC-AS3. Immunohistochemistry analysis revealed that the tumors developed from shHOXC-AS3 cells displayed lower Ki-67 staining than the control group. **b** Analysis of an experimental metastatic animal model was performed by injecting BGC-823 cells stably transfected with HOXC-AS3 knockdown into nude mice. Lungs from the mice in each experimental group, together with the number of tumor nodules on lung surfaces are shown. Visualization of the entire lung and HE-stained lung sections. *P < 0.05, **P < 0.01

To further determine whether HOXC-AS3 affects cell proliferation of GC in vivo, BGC-823 cells and SGC-7901 stably transfected with sh-HOXC-AS3 or control vector were inoculated into nude mice. Sixteen days after the injection, the tumors formed in the sh-HOXC-AS3 group were substantially smaller than those in the control group (Fig. 4a). Moreover, the mean tumor weight at the end of the experiment was markedly lower in the sh-HOXC-AS3 group compared with the control vector group (Fig. 4a). Tumors formed from stably sh-HOXC-AS3-transfected BGC-823 and SGC-7901 cells exhibited decreased positivity for Ki-67 than tumors from the control cells (Fig. 4a). These findings indicate that knockdown of HOXC-AS3 inhibits tumor growth in vivo.

To validate the effects of HOXC-AS3 on cell metastasis in vivo, BGC-823 cells stably transfected with sh-HOXC-AS3 or control vector were injected into the tail veins of nine mice. Metastatic nodules on the surface of the lungs were counted after 7 weeks. Ectopic knockdown of HOXC-AS3 reduced the number of metastatic nodules compared with the control group (Fig. 4b). This difference was further confirmed following examination of the entire lungs and by hematoxylin and eosin (HE) staining of lung sections (Fig. 4b). Our in vivo data, therefore, complemented the results of the functional in vitro studies involving HOXC-AS3.

### HOXC-AS3 interacts with YBX1

**Fig. 5:**
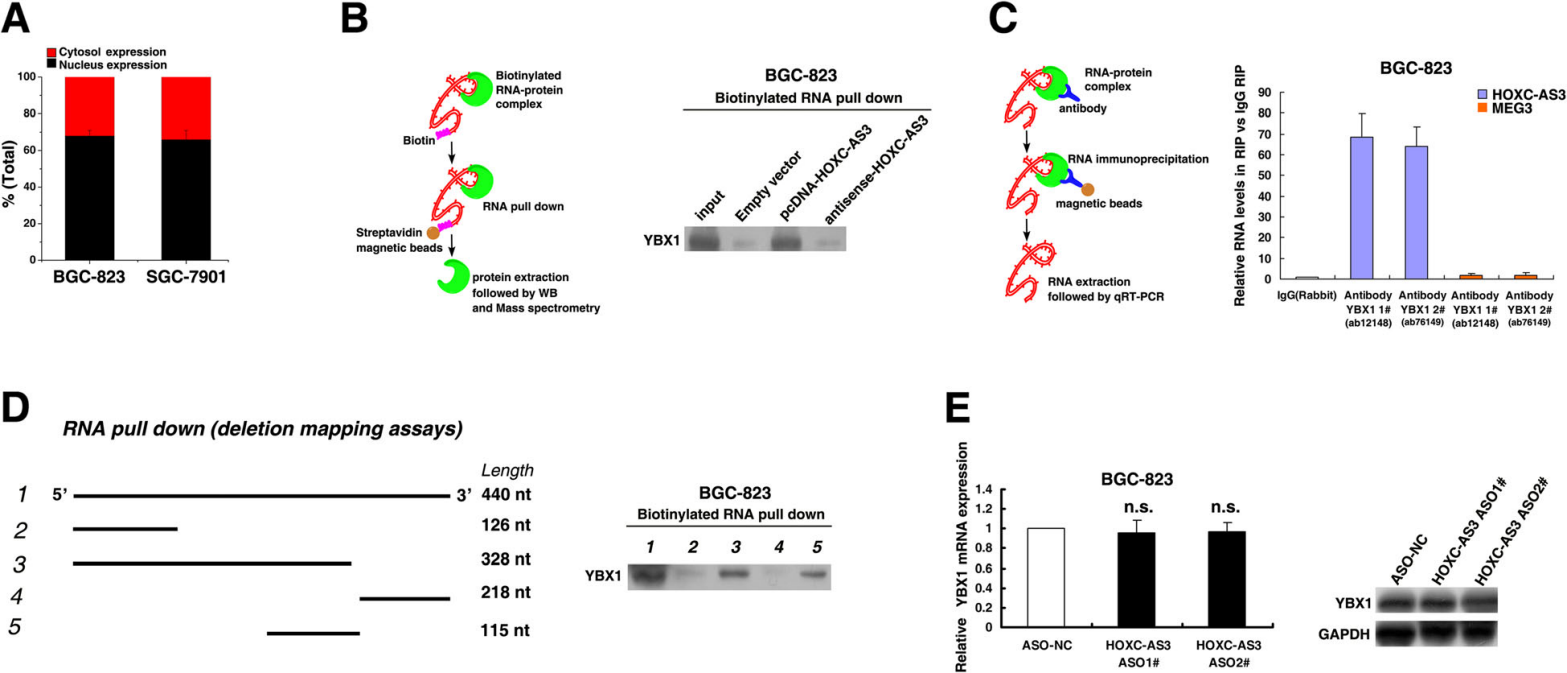
HOXC-AS3 Interacts with YBX1. **a** After nuclear and cytosolic separation, RNA expression levels were measured by qRT-PCR. GAPDH was used as a cytosol marker and U6 was used as a nucleus marker. **b** For the in vitro transcribed, pull-down assays-MS and WB assays showed that desthiobiotinylation-HOXC-AS3 could bind YBX1 in BGC-823 cells. **c** RIP experiments for YBX1 (two different antibodies from Abcam) were performed and the coprecipitated RNA was subjected to qRT-PCR for HOXC-AS3. The fold enrichment of HOXC-AS3 in RIPs is relative to its matching IgG control RIP. LncRNA MEG3 was a negative control. **d** Using a series of HOXC-AS3 deletion mapping constructs and RNA pull-down-WB assays, the YBX1-binding activity mapped to nucleotides 115 nt of HOXC-AS3. The profiles are established by RNA pull-down of the BGC-823 extract, and the retrieved proteins are detected by immunoblotting. **e** The qPCR and western blot assays detected the expression of YBX1 after knockdown of HOXC-AS3. *P < 0.05, n.s., not significant

To explore the mechanism of HOXC-AS3, firstly, we examined whether HOXC-AS3 acts *in cis*, affecting nearby gene expression (HOXC10; HOXC-AS3 was an antisense transcript of HOXC10). We found that knockdown of HOXC-AS3 did not affect expression of HOXC10 (data not shown), indicating it may act *in trans*. Then, subcellular fractionation location assays showed a considerable increase in HOXC-AS3 expression in the nucleus versus the cytosol (Fig. 5a), thus suggesting that HOXC-AS3 may play a major regulatory function at the transcriptional level, indicating it might interact with nucleus molecules or proteins. Then, we performed an RNA pull-down assay followed by a proteomic analysis of the HOXC-AS3-associated protein complex in BGC-823 cells (Fig. 5b). We incubated the in vitro transcribed HOXC-AS3 bound to beads with BGC-823 nuclear extracts to purify the HOXC-AS3 RNA-protein complex, and protein identity was revealed by mass spectrometry. Among the highly enriched proteins (Additional file 4: Table S3), only YBX1 was detected by western blotting from three independent RNA pull-down assays. YBX1 attracted our attention because of its established role in tumorigenesis and this notable protein was a known transcription factor [22, 23], with 12 unique peptides detected in this MS analysis. To further validate the physical interaction between HOXC-AS3 and YBX1, we performed RNA pull-down followed by western blotting with YBX1 antibodies. Our results showed that labeled HOXC-AS3 RNA, but not empty vector or an antisense HOXC-AS3, specifically retrieved YBX1 from BGC-823 cell extracts (Fig. 5b). We also performed RNA immunoprecipitation (RIP) for the RNA-YBX1 complex using different YBX1 antibodies and measured the amount of HOXC-AS3 associated with YBX1 immunoprecipitates but not MEG3. LncRNA MEG3 was a negative control (Fig. 5c). Using a series of HOXC-AS3 deletion mapping, the YBX1-binding activity mapped to nucleotides 115 nt of HOXC-AS3 (Fig. 5d). Additionally, the qRT-PCR and western blots showed that knockdown of HOXC-AS3 could have had no effect on the expression of YBX1 (Fig. 5e). Our results proved that HOXC-AS3 could bind to YBX1 but have no effect on YBX1 expression. These results indicated that HOXC-AS3 may participate in the tumorigenesis of GC through transcriptional regulation of other genes via binding to YBX1.

### A large set of genes that are linked to cell proliferation and cell migration were coregulated by interaction of HOXC-AS3 and YBX1

**Fig. 6:**
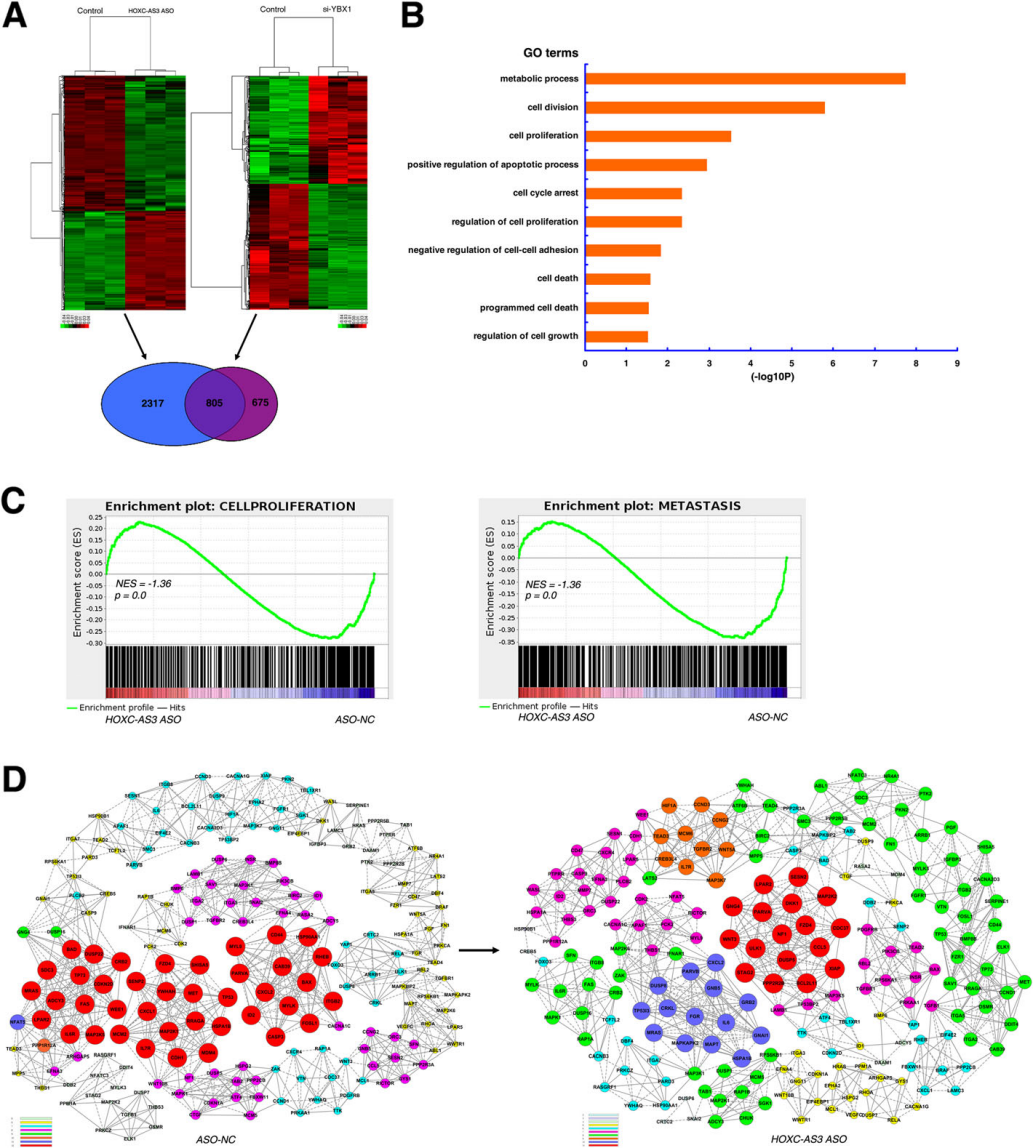
Downstream gene network of HOXC-AS3 and a common set of target genes shared by HOXC-AS3 and YBX1. **a** Mean-centered, hierarchical clustering of gene transcripts altered (≥ 1.5-fold change) after knockdown of HOXC-AS3 and YBX1 in BGC-823 cells, with three replicates. **b** Gene ontology analysis for all genes with altered expression after knockdown of HOXC-AS3. **c** GSEA showed that genes in response to HOXC-AS3 knockdown are enriched for the gene sets significantly related to cell proliferation and metastasis. **d** Coexpression-network analysis of differentially expressed genes for knockdown of HOXC-AS3. The solid lines represent positive Pearson’s correlation, whereas the dotted lines represent negative Pearson’s correlation. The node size and node color represent the coexpression ability k-core of gene, the greater the node size, the greater the k-core value

To ascertain the mechanism of action of HOXC-AS3/YBX1*-*driven oncogenesis of GC and to determine the gene expression changes downstream of HOXC-AS3, we evaluated the global effects of HOXC-AS3 knockdown compared with those of YBX1 knockdown using RNA transcriptome sequencing (Fig. 6a, Additional file 5: Table S4 and Additional file 6: Table S5). A total of 3122 genes were significantly upregulated or downregulated in BGC-823 cells as a consequence of HOXC-AS3 knockdown (fold change ≥ 1.5) (Fig. 6a). For RNA-seq results of HOXC-AS3 knockdown, gene ontology (GO) analysis showed that the most significantly overrepresented biological processes included pathways involved in cell division, cell proliferation, and cell death (Fig. 6b). Gene set enrichment analysis (GSEA) revealed that the gene sets were significantly related to cell proliferation and metastasis (Fig. 6c). We also built a coexpression network based on the differentially expressed genes that are functionally related. The co-expression network was constructed according to the gene normalized expression (RPKM), and the k-core was calculated based on Pearson’s relationship to represent the core status of each gene in different groups. The k-core factor was then applied to identify key regulatory genes, which likely play pivotal roles in gene interactions and regulation (Fig. 6d), including for key cancer genes, such as p21, FAS, CCND1, and CDK2; real-time PCR confirmed these genes after knockdown of HOXC-AS3 (Additional file 3: Figure S2B). Additionally, a total of 1480 genes were significantly upregulated or downregulated in BGC-823 cells as a consequence of YBX1 knockdown (fold change ≥ 1.5) (Fig. 6a and Additional file 3: Figure S2A). Intriguingly, the expression of 805 of the 3122 HOXC-AS3-regulated genes was also regulated by knockdown of YBX1 (Fig. 6a). The high overlap between HOXC-AS3 and YBX1-mediated regulation strongly indicates a functional interaction between HOXC-AS3 and YBX1. YBX1 functions as an RNA-binding protein and has been implicated in numerous cellular processes, including the regulation of transcription and translation [24]. YBX1 was shown to be a protein with a nucleic acid-binding common domain in gene promoter, CCAAT-box, which is a high consensus sequence in eukaryotes [25]. Moreover, YBX1 serves as a transcriptional activator and YBX1 activation was associated with cancer progression, including gastric cancer [23, 26, 27]. Our results showed that YBX1 was significantly upregulated in 60 pairs of GC tissues by qRT-PCR analysis. Knockdown of YBX1 could significantly inhibit cell proliferation and migration (Additional file 3: Figure S2C). Previous research supports a role for lncRNA-YBX1 interactions as mediators of gene transcriptional regulation [28]. In particular, 476 genes were corepressed by knockdown of HOXC-AS3 and YBX1. These results indicated that a common set of target genes linked to cell proliferation and cell migration were shared by HOXC-AS3 and YBX1.

**Fig. 7:**
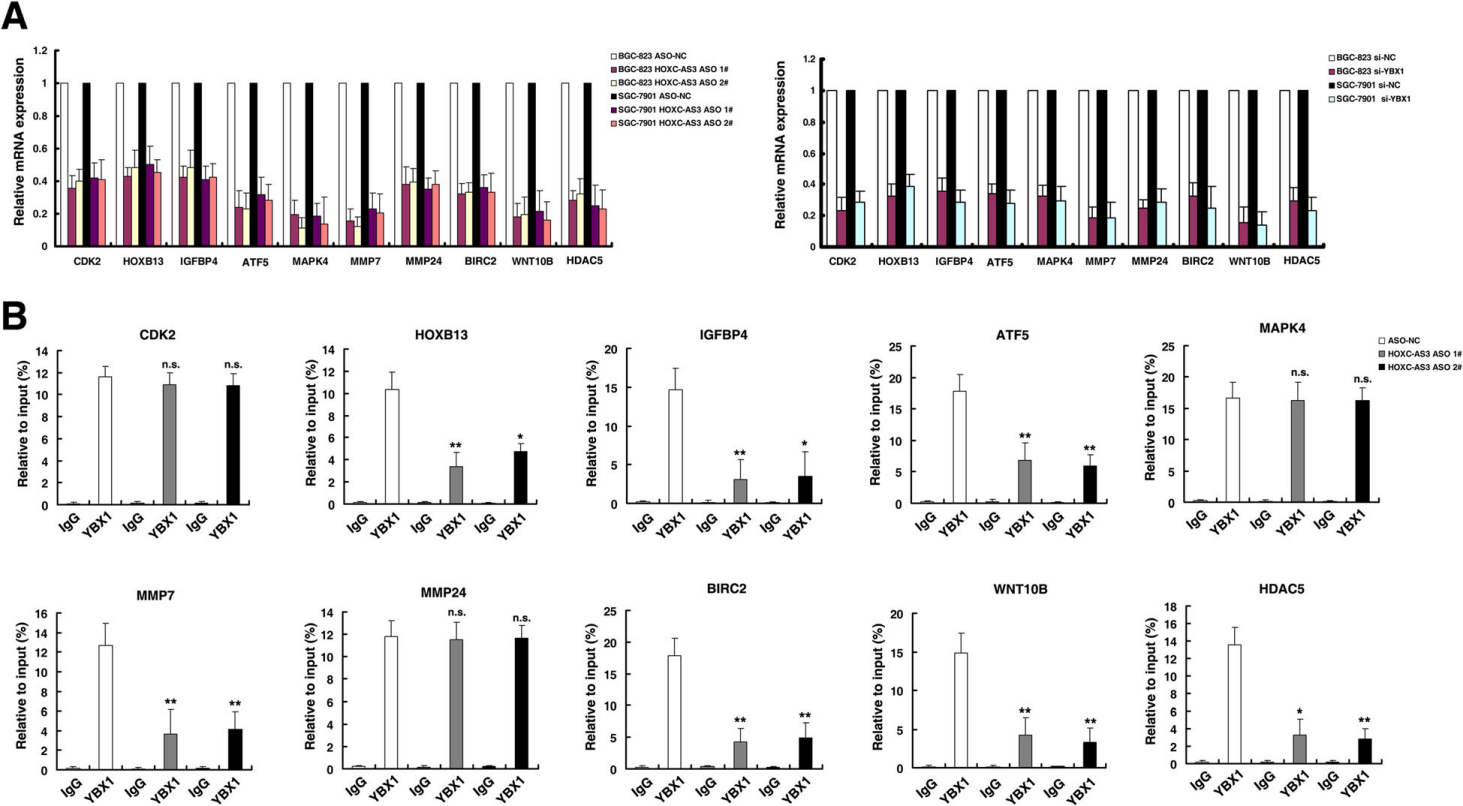
HOXC-AS3 regulates the transcription of a large set of genes through an interaction with YBX1. **a** The altered mRNA levels of genes were selectively confirmed by qRT-PCR in knockdown HOXC-AS3 and YBX1. **b** ChIP-qPCR of YBX1 of the promoter region of these gene loci after knockdown HOXC-AS3 in BGC-823 cells. Antibody enrichment was quantified relative to the amount of input DNA. Antibody directed against IgG was used as a negative control. *P < 0.05, **P < 0.01. n.s., not significant

Genes were corepressed by knockdown of HOXC-AS3/YBX1. They included many well-known proliferation and migration-related genes (e.g., CDK2, HOXB13, IGFBP4, ATF5, MAPK4, MMP7, MMP24, BIRC2, WNT10B, and HDAC5). These genes were selectively confirmed by qRT-PCR after knockdown of HOXC-AS3 and YBX1 in BGC-823 and SGC-7901 cells (all these genes containing CCAAT-box, YBX1-binding site in their promoters) (Fig. 7a). Given that YBX1 regulates gene transcription by binding to the promoter regions of target genes, we examined whether HOXC-AS3 knockdown affected YBX1 occupancy of the promoter regions in these target genes. Then ChIP assays followed by qPCR demonstrated that knockdown of HOXC-AS3 decreased the binding of YBX1 levels across the promoters of the most coregulated genes (Fig. 7b; among the ten target genes tested, YBX1 occupancy were indeed affected in the promoter regions of seven genes following knockdown of HOXC-AS3). These findings demonstrate that HOXC-AS3 is important in regulating gene transcription expression, presumably in part by regulating the association between YBX1 and the promoter regions of its target genes.

### HOXC-AS3 regulates the expression of HDAC5

**Fig. 8:**
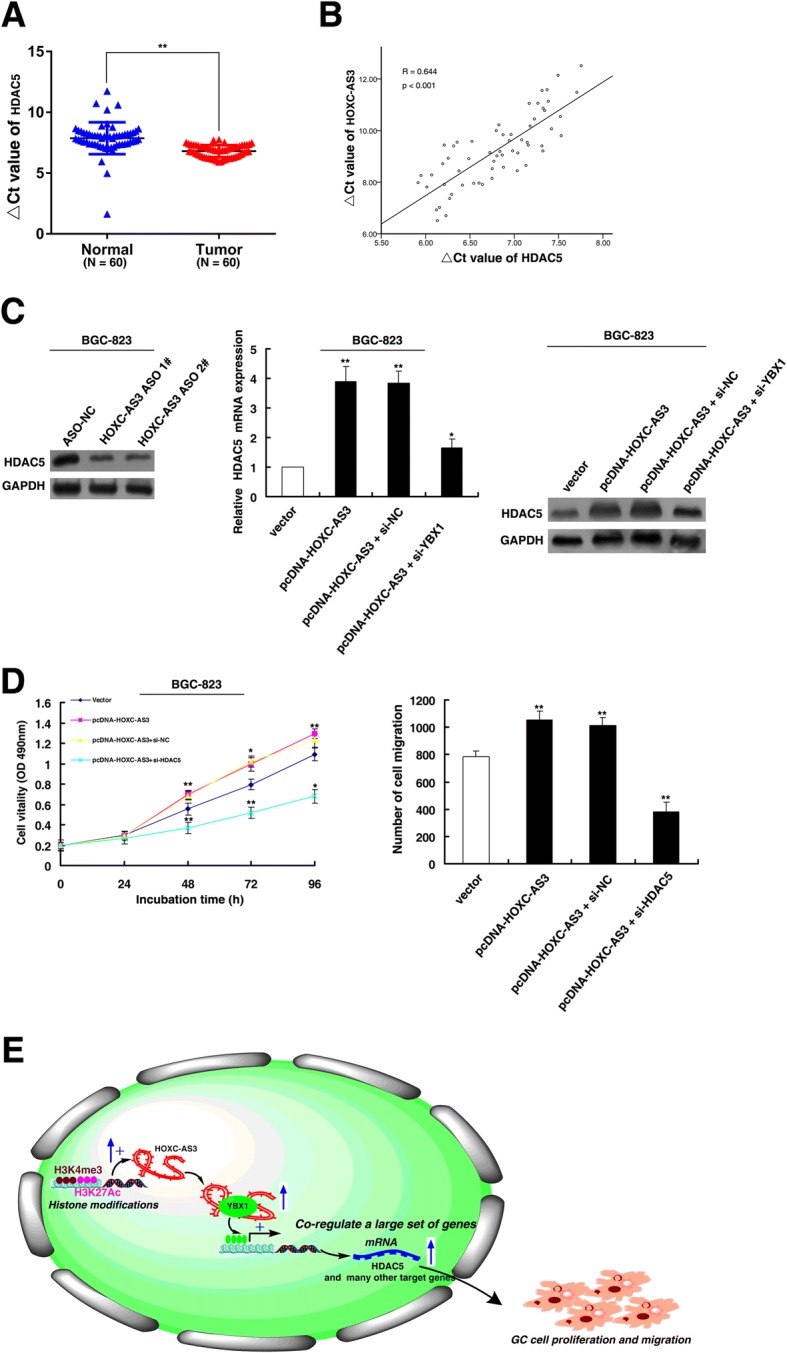
HOXC-AS3 regulates expression of HDAC5. **a** Based on qRT-PCR assays, the level of HDAC5 was upregulated in 60 pairs GC tissues. The ΔCt value was determined by subtracting the GAPDH Ct value from the HDAC5 Ct value. A smaller ΔCt value indicates higher expression. **b** The level of HOXC-AS3 in GC tissues showed a significant positive correlation with the relative level of HDAC5 expression (*n* = 60). **c** Western blot assays detected the expression of HDAC5 after knockdown of HOXC-AS3 in BGC-823 cells. The promotion of HDAC5 (mRNA and protein) by HOXC-AS3 was significantly reversed by knockdown of YBX1, based on qPCR and western blot assays. **d** MTT assays showed that knockdown of HDAC5 could suppress cell proliferation, and knockdown of HDAC5 could also reverse HOXC-AS3-mediated growth promotion. **e** Proposed model in which HOXC-AS3 mediates the proliferation and migration progression of GC

Among the common targets of HOXC-AS3 and YBX1, HDAC5 is of particular interest because of its remarkable expression fold change upon HOXC-AS3 knockdown (Fig. 7a) and its significant contribution to tumorigenesis [29]. Aberrant activation of HDAC5 in tumor cells leads to dysregulation of a diverse set of genes mainly involved in the regulation of proliferation, migration, and angiogenesis [30]. Our results showed that HDAC5 was significantly upregulated in 60 pairs of GC tissues. Further analysis demonstrated that HOXC-AS3 was positively correlated with HDAC5 expression in GC tissues (Fig. 8a, b). Then, we verified the results at the protein level for HDAC5 after knockdown of HOXC-AS3 (Fig. 8c). Furthermore, the increase of HDAC5 by HOXC-AS3 was reversed with treatment by YBX1 siRNAs at both the mRNA and protein levels (Fig. 8c). Moreover, knockdown of HDAC5 induced the suppression of proliferation and migration in BGC-823 cells (Fig. 8d and Additional file 3: Figure S2D). In addition, knockdown of HDAC5 could reverse HOXC-AS3-mediated growth and migration promotion (Fig. 8d). Together, these results demonstrate that HOXC-AS3 exerts its function, at least in part by regulating HDAC5 expression.

## Discussion

Before the discovery of noncoding RNAs, explorations for cancer drivers focused on protein-coding genes that resided in recurrent alterations in cancer genomes. However, the newly discovered lncRNAs have emerged as important players in cellular development and human diseases, especially in cancer. In the present study, utilizing publicly available lncRNA expression profiling data of gastric cancer and integrating analyses of TCGA data, we screened and identified a novel lncRNAs HOXC-AS3. The high expression of HOXC-AS3 in GC patients was positively correlated with advanced TNM stage. Moreover, high HOXC-AS3 expression in GC tissues was associated with a poor prognosis and could be an independent prognostic indicator. In addition, GTEx data (https://www.gtexportal.org/) showed that HOXC-AS3 had lower basal expression in normal GC tissues (Additional file 3: Figure S1B). This further demonstrates an important role of HOXC-AS3 in the carcinogenesis of GC. These results and our functional evidence for HOXC-AS3 suggested that HOXC-AS3 might exhibit an important role in GC progression.

HOXC-AS3, which is located at chromosome 12q13.13, was an antisense transcript of HOXC10. HOX genes are essential for morphogenesis and development [31, 32]. The dysregulation of HOX gene expression has been shown in many diverse cancers [33, 34], and lncRNA generation in HOX genes may play important roles in tumorigenesis. For example, as a well-known lncRNA, HOTAIR, also located at chromosome 12q13.13, is an antisense transcript of HOXC11 and was an oncogenic lncRNA in many different types of cancer [35]. Our previous study also found that a lncRNA of the HOX gene family, HOXA11-AS, could play an important role in GC tumorigenesis [14]. In our present study, we found a novel lncRNA in the HOX genes family. Our results revealed that gain of H3K4me3 and H3K27Ac activation of the promoter also partly contributed to activation of HOXC-AS3 in GC, both in cells and tissues. Similar to protein coding transcripts, the transcription of lncRNAs is subject to typical epigenetics-mediated and transcription factor-mediated regulation. For example, the lncRNA MEG3 was lost in tumors due to an increase in CpG methylation within the promoter [36]. Histone deacetylase3-suppressed the lncRNA LET in hepatocellular carcinoma by reducing the histone acetylation-mediated modulation of the promoter region [37].

In our study, we found that inhibition of HOXC-AS3 repressed GC proliferation and migration both in vitro and in vivo*.* RNA-seq found that knockdown of HOXC-AS3 affected key cancer-related genes, such as p21, FAS, and CCND1. Mechanistic investigations found that HOXC-AS3 could bind to YBX1, but not affect YBX1 expression. These results indicated that HOXC-AS3 may participate in the tumorigenesis of GC through the transcriptional regulation of other genes via binding to YBX1 in *trans*. To probe the HOXC-AS3-associated pathway on an unbiased basis in the tumorigenesis of GC, RNA-Seq assays were used after simultaneous knockdown HOXC-AS3 and YBX1. Notably, loss of HOXC-AS3 in GC cells recapitulated the phenotype observed after YBX1 knockdown. In addition, a significant fraction of the genes regulated by HOXC-AS3 loss were similarly regulated by loss of YBX1. Thus, HOXC-AS3 may act, in part, by regulating the interaction between YBX1 and the promoter of target genes, although HOXC-AS3 likely interacts with other RNA-binding proteins that will need to be identified to fully understand its molecular function. YBX1 was reported to play a role in regulating cell signaling, transcription, and tumorigenesis [23]. Furthermore, YBX1 serves as a transcriptional activator and regulates much gene transcription [38]. YBX1 was a protein with a nucleic acid-binding common domain in the gene promoter, CCAAT-box, which is a high consensus sequence in eukaryotes. We found that YBX1 is overactive in GC and knockdown of YBX1 inhibits the proliferation of GC cells. Our results showed that HOXC-AS3 could bind to YBX1, thus transcriptionally regulating a large set of genes that are linked to cell proliferation and cell migration in gastric cancer cells, such as MMP7, WNT10B, and HDAC5, thus promoting GC cell proliferation and migration.

The HDAC5 gene is a member of the histone deacetylase (HDAC) from a family of enzymes. Histone acetylation and deacetylation play important roles in chromatin remodeling and gene expression. An imbalance of these reactions leads to the growth, migration, and apoptosis of cancer cells. Histone deacetylase (HDAC) inhibitors were shown to have antitumor effects in clinical trials [39, 40]. Over-activation of HDAC5 was found in many different types of cancer [41–43]. A previous study showed that HDAC5 was induced in gastric cancer cells [39]. Here, we also provide evidence for high expression of HDAC5 in gastric cancer, and knockdown of HDAC5 inhibits the proliferation of GC cells. We also found that the transcriptional activation of HDAC5 is partly mediated by HOXC-AS3 in the tumor progression of GC through binding to YBX1, thus facilitating GC cell proliferation and migration. In addition to HDAC5, there are many other important genes related to tumorigenesis, and they are also regulated by HOXC-AS3 in a similar manner.

## Conclusions

In summary, abnormal histone modification**-**mediated activation of a novel lncRNA HOXC-AS3 promotes GC cell proliferation and migration through transcriptional activation of a large set of genes through an interaction with YBX1. Our data reveal a role for HOXC-AS3 in GC tumorigenesis and may provide a strategy for using HOXC-AS3 as a potential biomarker and a therapeutic target for patients with GC (Fig. 8e).

## Methods

### Tissue collection and ethics statement

A total of 112 patients in this study underwent resection of the GC at The Affiliated Jiangyin Hospital of Southeast University Medical College, affiliated Xuzhou Central Hospital of Southeast University Medical College. The study was approved by the Medical Ethical Committee of Southeast University Medical College (Nanjing, Jiangsu, PR China), and it was performed in compliance with the Helsinki Declaration. All patients have given written informed consent for publication. The clinicopathological characteristics of the GC patients are summarized in Additional file 1: Table S1.

### Gastric cancer RNA-expression data retrieval and analysis

#### Microarray data analysis

Microarray datasets from the GEO database were used to test HOXC-AS3 differential expression. Raw microarray data was downloaded from GEO including GSE50710 and GSE58828. Then, the raw microarray data were normalized and z-score-transformed using RMAExpress (http://www.rmaexpress.bmbolstad.com/). RNA-Seq data (from TCGA) of lncRNAs of gastric cancer were from TANRIC (http://ibl.mdanderson.org/tanric/_design/basic/index.html) [21].

### RACE (rapid amplification of cDNA ends)

5′-RACE, 3′-RACE, and full-length amplification of HOXC-AS3 were performed using the SMART RACE cDNA Amplification Kit (Cat. 634858, Clontech, Palo Alto, CA, USA) according to the manufacturer’s instructions. The gene-specific primers used for RACE analysis were presented in Additional file 7: Table S6.

### Transfection of cell lines

LNA-ASO (Locked Nucleic Acid, antisense oligonucleotide) targeting HOXC-AS3 and negative control LNA-ASO were designed and synthesized by Exiqon (Exiqon, Vedbaek, Denmark). GC cells were transfected with the LNA-ASOs using Oligofectamine transfection reagent (RNAi MAX, Invitrogen) according to the manufacturer’s instructions. Cells were harvested for analyses 48 h after transfections. The sequences of ASO were listed in Additional file 7: Table S6. The sequences for siRNAs were listed in Additional file 7: Table S6. Scrambled negative control siRNA was purchased from Invitrogen (Invitrogen, CA, USA). The interference target sequence of YBX1 was acquired according to a previous study [44]. The HDAC5 siRNA was from Santa Cruz (sc-35542). The plasmid was transfected into GC cells using the X-tremeGENE™ HP DNA Transfection Reagent (Roche) according to the manufacturer’s instructions.

### Subcellular fractionation location

Separation of the nuclear and cytosolic fractions was performed using the PARIS Kit (Cat. AM1921, Invitrogen, CA, USA) according to the manufacturer’s instructions.

### In vitro transcription assays and RNA pull-down mass spectrometry (LC-MS/MS) assays

In vitro translation assays were performed using mMESSAGE mMACHINE™ T7 Transcription Kit according to the manufacturer’s instructions (Cat. AM1344, Invitrogen, CA, USA). Then, HOXC-AS3 RNAs were labeled with desthiobiotinylation using the Pierce RNA 3′ End Desthiobiotinylation Kit (Cat. 20164, Magnetic RNA-Protein Pull-Down Kit, Components, Thermo). RNA pull-down assays were performed with Pierce Magnetic RNA-Protein Pull-Down Kit according to the manufacturer’s instructions (Cat. 20164, Magnetic RNA-Protein Pull-Down Kit, Thermo). After elution of lncRNA-interacting proteins, they were subjected to mass spectrometric analysis. LC**-**MS/MS experiments were performed with an LTQ linear ion trap mass spectrometer (Thermo Finnigan, San Jose, CA) equipped with a microspray source.

### RNA immunoprecipitation (RIP) assays

RNA immunoprecipitation (RIP) experiments were performed using a Magna RIP™ RNA-Binding Protein Immunoprecipitation Kit (Cat. 17-701, Millipore, USA) according to the manufacturer’s instructions. The antibodies for RIP assays of YBX1 (Cat. ab12148, ab76149) were from Abcam.

### Transcriptome sequencing

Total RNA from BGC-823 cells with HOXC-AS3/YBX1 knockdown and control cells were isolated and quantified. The concentration of each sample was measured with a NanoDrop 2000 (Thermo Scientific, USA). The quality was assessed by an Agilent2200 (Agilent, USA). The sequencing library of each RNA sample was prepared using the Ion Proton Total RNA-Seq Kit v2 according to the protocol provided by the manufacturer (Life Technologies, USA). Data are available in Additional file 5: Table S4 and Additional file 6: Table S5**.**

### Chromatin immunoprecipitation (ChIP) assays

ChIP assays were performed using the EZ-CHIP KIT according to the manufacturer’s instruction (Cat. 17-408, Millipore, USA). The antibodies for Histone H3, acetyl-histone H3 Lys27 (H3K27Ac, Cat. ab4729), and H3 trimethyl Lys4 (H3K4me3, Cat. ab8580) were from Abcam. The ChIP primer sequences were listed in Additional file 7: Table S6. The antibody for YBX1 (Cat. ab12148) was from Abcam. Quantification of immunoprecipitated DNA was performed using qPCR. ChIP data was calculated as a percentage relative to the input DNA from the equation 2^[Input Ct − Target Ct]^ × 100 (%).

### Statistical analysis

All statistical analyses were performed using SPSS 20.0 software (IBM, SPSS, USA). The significance of differences between groups was estimated by Student’s *t* test, *χ*^2^ test, or Wilcoxon test, as appropriate. OS rates were calculated by the Kaplan–Meier method with the log-rank test for comparison. Survival data were evaluated using univariate and multivariate Cox proportional hazards model. Variables with a value of *P* < 0.05 in univariate analysis were used in the subsequent multivariate analysis based on the Cox regression analyses. Two-sided *P* values were calculated, and a probability of 0.05 was selected for statistical significance.

Additional methods are described in Additional file 8: Supplementary Methods.

## Additional files

Additional file 1: **Table S1.**The clinic-pathological factors of 112 GC patients. (XLS 10 kb)

Additional file 2: **Table S2.** Univariate and multivariate analyses of clinicopathologic factors for overall survival in 112 patients with GC. (XLS 10 kb)

Additional file 3: **Figure S1.** (A) HOXC-AS3 expression after ASO-mediated knockdown and plasmid-mediated overexpression in GC cells. (B) Expression of HOXC-AS3 across diverse normal human tissues from GTEx. **Figure S2.** (A) Western blots were performed to detect YBX1 expression. (B) The altered mRNA levels of genes were confirmed by qRT-PCR for knockdown HOXC-AS3 in BGC-823 and SGC-7901 cells. (C) Based on qRT-PCR assays, the level of YBX1 was upregulated in 60 pairs GC tissues. MTT assays and transwell assays were used to investigate the changes in proliferation and migratory abilities of BGC-823 cells after transfection. (D) Western blots were performed to detect HDAC5 expression after transfection in BGC-823 cells. (DOC 1991 kb)

Additional file 4: **Table S3.** A list of the top ten potential HOXC-AS3-interacting protein candidates in BGC-823 cells based on RNA-protein pull-down assays and mass spectrometry analysis. (XLS 15 kb)

Additional file 5: **Table S4.** The mRNA variation abundance (≥1.5-fold) for HOXC-AS3-knockdown in BGC-823 cells. (XLS 491 kb)

Additional file 6: **Table S5.** The mRNA variation abundance (≥1.5-fold) for YBX1-knockdown in BGC-823 cells. (XLS 236 kb)

Additional file 7: **Table S6.** The list of primers and siRNA /ASO sequence. (XLS 20 kb)

Additional file 8 Supplementary Methods. (DOC 44 kb)
