# Supplementary material for: A novel long noncoding RNA HOXC-AS3 mediates tumorigenesis of gastric cancer by binding to YBX1
Source: Genome Biol. 2018 Oct 4;19:154. doi: 10.1186/s13059-018-1523-0 (PMC6172843; doi:10.1186/s13059-018-1523-0)
Supplement: Supplementary file 3 — Figure S1. (A) HOXC-AS3 expression after ASO-mediated knockdown and plasmid-mediated overexpression in GC cells. (B) Expression of HOXC-AS3 across diverse normal human tissues from GTEx. Figure S2. (A) Western blots were performed to detect YBX1 expression. (B) The altered mRNA levels of genes were confirmed by qRT-PCR for knockdown HOXC-AS3 in BGC-823 and SGC-7901 cells. (C) Based on qRT-PCR assays, the level of YBX1 was upregulated in 60 pairs GC tissues. MTT assays and transwell assays were used to investigate the changes in proliferation and migratory abilities of BGC-823 cells after transfection. (D) Western blots were performed to detect HDAC5 expression after transfection in BGC-823 cells. (DOC 1991 kb) [file 13059_2018_1523_MOESM3_ESM.doc]

**
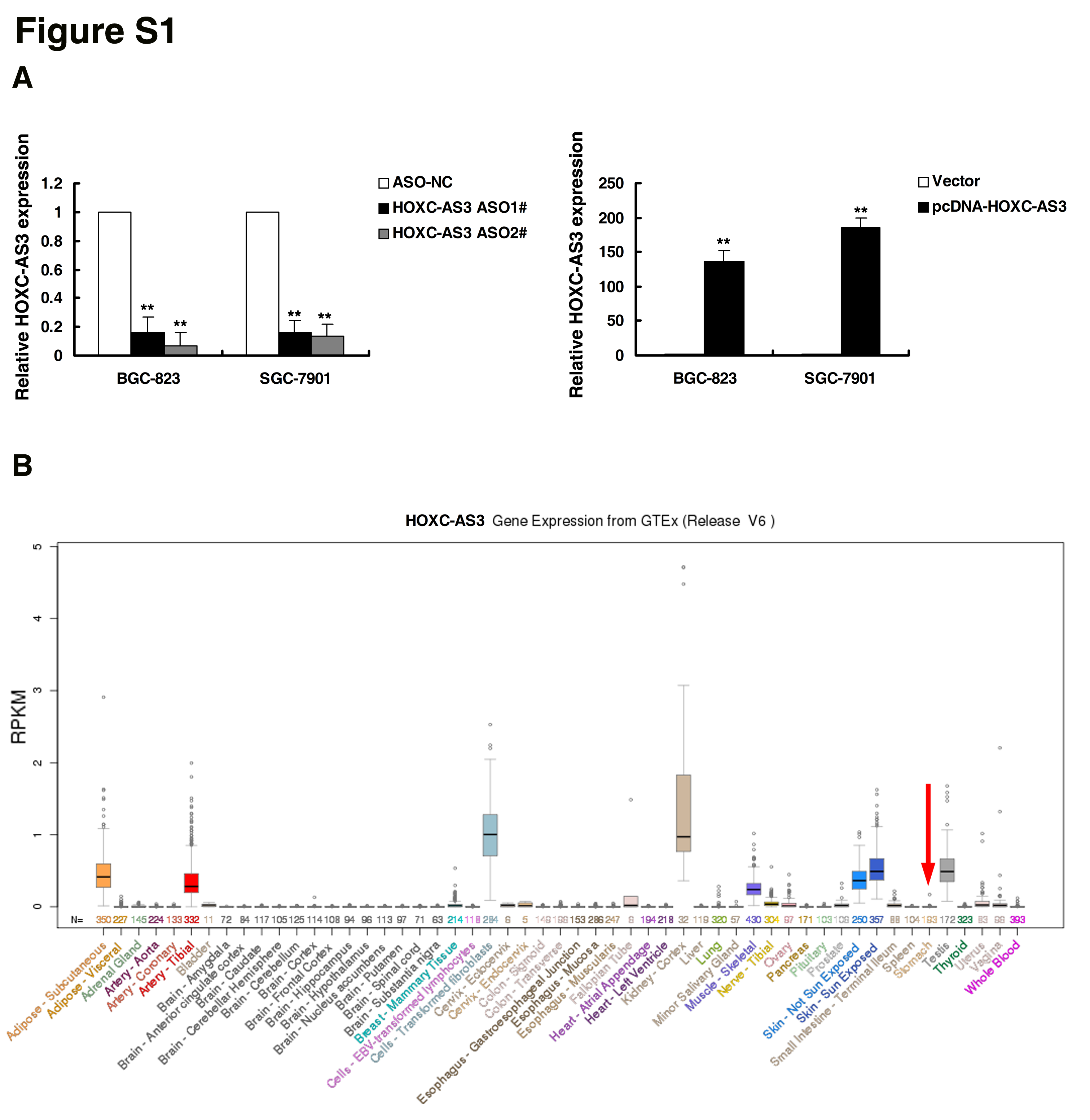
**

**Figure S1** (A) The qRT-PCR was performed to detect HOXC-AS3 expression after ASO-mediated knockdown and plasmid-mediated overexpression in BGC-823 and SGC-7901 cells. (B) Expression of HOXC-AS3 across diverse normal human tissues from GTEx (<https://www.gtexportal.org/>).

**
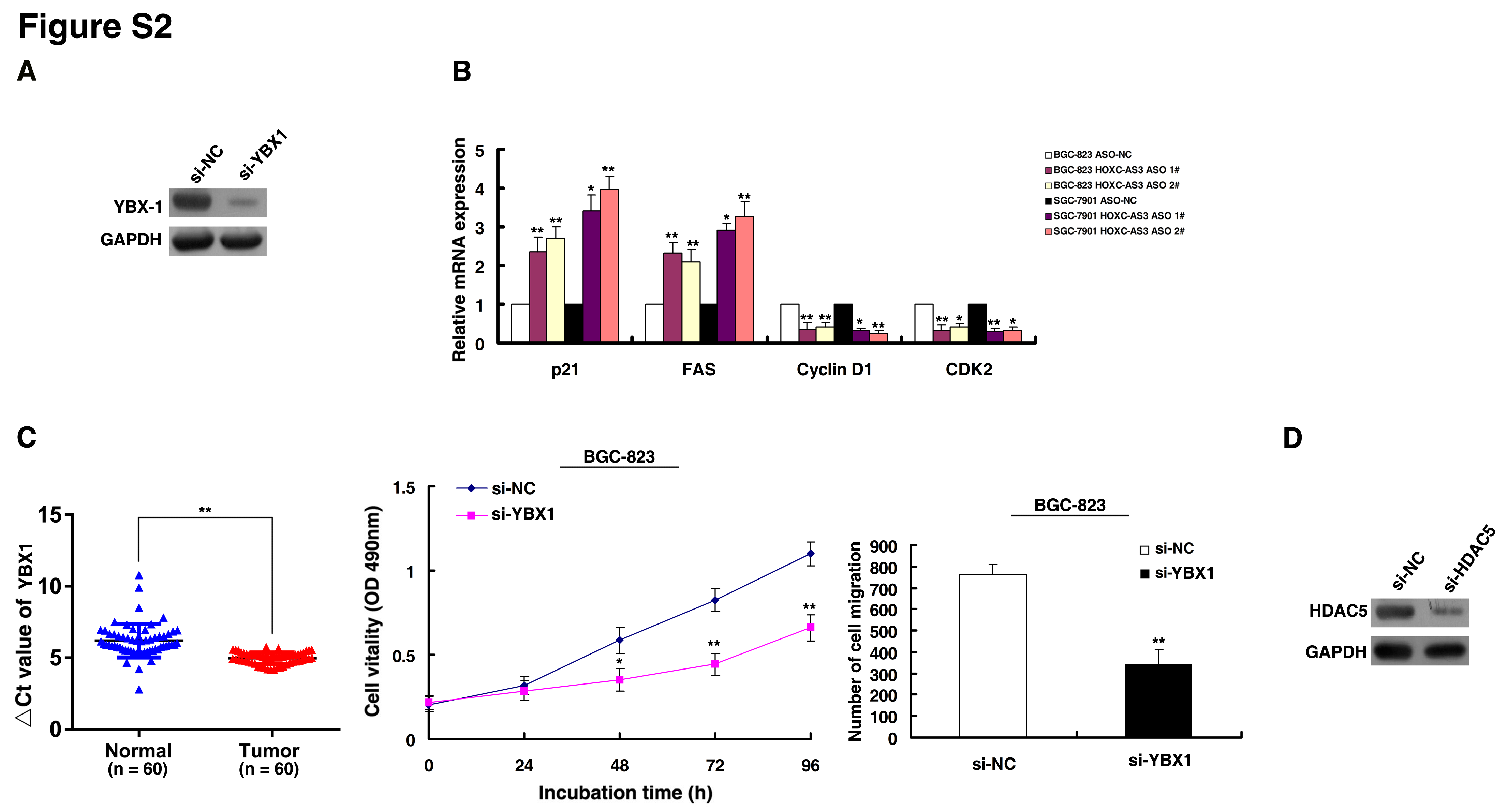
**

**Figure S2** (A) Western blots were performed to detect YBX1 expression after siRNA transfection in BGC-823 cells. (B) The altered mRNA levels of genes were confirmed by qRT-PCR for knockdown HOXC-AS3 in BGC-823 and SGC-7901 cells. (C) Based on qRT-PCR assays, the level of YBX1 was upregulated in 60 pairs GC tissues. The ΔCt value was determined by subtracting the GAPDH Ct value from the HDAC5 Ct value. A smaller ΔCt value indicates higher expression. MTT assays and transwell assays were used to investigate the changes in proliferation and migratory abilities of BGC-823 cells after transfection, respectively. (D) Western blots were performed to detect HDAC5 expression after siRNA transfection in BGC-823 cells. *P<0.05, **P<0.01.
