# Supplementary material for: A novel long noncoding RNA HOXC-AS3 mediates tumorigenesis of gastric cancer by binding to YBX1
Source: Genome Biol. 2018 Oct 4;19:154. doi: 10.1186/s13059-018-1523-0 (PMC6172843; doi:10.1186/s13059-018-1523-0)
Supplement: Supplementary file 8 — Supplementary Methods. (DOC 44 kb) [file 13059_2018_1523_MOESM8_ESM.doc]

**Supplementary Methods**

**RNA extraction and qRT-PCR analyses**

Total RNA was extracted from tissues or cultured cells using TRIzol reagent (Invitrogen, Carlsbad, CA). For qRT-PCR, RNA was reverse transcribed to cDNA by using a Reverse Transcription Kit (Takara, Dalian, China). Real-time PCR analyses were performed with SYBR Green (Takara, Dalian China). Results were normalized to the expression of GAPDH. The rest of primers were listed in **Supplementary Table S6**.

**Probe ID annotation and prognostic analysis**

1. Launch the <https://www.ncbi.nlm.nih.gov/geoprofiles> (GEO Profiles) website and input “HOXC-AS3”.
2. In the searching result, you can find “Reporter” type displaying the information as following: GPL570, 1561273_at (ID_REF), GDS5324, 100874365 (Gene ID), BC035260.
3. 1561273_at (ID_REF) was the probe ID for GPL570 platform which is known as the Affymetrix Human Genome U133 Plus 2.0 Array.
4. Launch the <http://kmplot.com/analysis/> website and then input “1561273_at” for starting KM Plotter prognostic analysis for gastric cancer, then drawing Kaplan-Meier plot.

**Cell culture**

GC cell lines were cultured in DMEM (GIBCO-BRL) and 1640 medium supplemented with 10 % fetal bovine serum (10 % FBS), 100 U/ml penicillin, and 100 mg/ml streptomycin in humidified air at 37 °C with 5% CO2.

**Plasmid constructs**

HOXC-AS3 cDNA was synthesized and cloned into the expression vector pcDNA3.1 (Invitrogen) according to the sequence of RACE. The HOXC-AS3 fragments for RNA pull down assays were then used as a template for generating constructs carrying deletions using respective primers. All PCR products were verified by DNA sequencing.

**Cell proliferation analysis**

Cell viability was tested with MTT kit (Sigma) according to the manufacturer’s instruction. For colony formation assay, a certain number of transfected cells were placed in each well of 6-well plates and maintained in proper media containing 10% FBS for two weeks, during which the medium was replaced every 4 days. Colonies were then fixed with methanol and stained with 0.1% crystal violet (Sigma) in PBS for 15 minutes. Colony formation was determined by counting the number of stained colonies. EdU experiments were performed using a EdU Cell Proliferation Assay Kit (Cat.C10310-1, Ruibo, Guangzhou, China) according to the manufacturer’s instructions.

**Cell migration assays**

For the migration assays, after transfection, 5 × 10^4^ cells in serum-free media were placed into the upper chamber of an insert (8-μm pore size; Millipore, Billerica, MA, USA). Medium containing 10% FBS was added to the lower chamber. After incubation for 24 h, the cells remaining on the upper membrane were removed with cotton wool. Cells that had migrated through the membrane were stained with methanol and 0.1% crystal violet, imaged, and counted using an IX71 inverted microscope (Olympus, Tokyo, Japan). Experiments were independently repeated three times.

**Western blot assay and antibodies**

The treated cells were lysed using mammalian protein extraction reagent RIPA (Beyotime, Haimen, China) supplemented with protease inhibitors cocktail (Roche) and PMSF (Roche). Cells protein lysates were separated by 10% SDS-polyacrylamide gel electrophoresis (SDS-PAGE) transferred to 0.22μm NC membranes (Sigma) and incubated with specific antibodies. Autoradiograms were quantified by densitometry (Quantity One software; Bio-Rad). GAPDH antibody was used as control. Anti-YBX1 (Dilution ratio, 1:2000, ab76149) and Anti-HDAC5 (Dilution ratio, 1:2000, ab1439) were from Abcam (Hong Kong, China).

***In vivo*** **assay**

Athymic male mice were purchased from the Animal Center of the Chinese Academy of Science (Shanghai, China) and maintained in laminar flow cabinets under specific pathogen-free conditions. For cell proliferation assay in *vivo*, BGC-823 and SGC-7901 cells were stably transfected with shRNA and empty vector and harvested from cell culture plates, then cells were xenografted into BALB/c male nude mice. The tumor volumes and weights were measured every 2 days in mice; the tumor volumes were measured as length×width^2^×0.5. Sixteen days after injection, the mice were killed and tumor weights were measured and used for further analysis. BGC-823 cells were stably transfected with shRNA and empty vector and harvested from cell culture plates, washed with PBS, and re-suspended at 2 × 10^7^ cells/ml. Suspended cells (0.1 ml) were injected into the tail veins of nine mice, which were sacrificed 7 weeks after injection. The lungs were removed and photographed, and visible tumors on the lung surface were counted. This study was carried out in strict accordance with the Guide for the Care and Use of Laboratory Animals of the National Institutes of Health. Our protocol was approved by the Committee on the Ethics of Animal Experiments of Southeast University.

**Gene Set Enrichment Analysis (GSEA) and co-expression Analysis**

The DEGs (differentially expressed gene) response to HOXC-AS3 knockdown was used for GSEA analysis in the GSEA tool[^1^](#_ENREF_1). Normalized enrichment score was calculated to compare enrichment analysis results across gene sets. Gene co-expression Networks were built according to the normalized expression values of genes selected from genes analyzed by the GSEA gene set analysis. RPKM value (Reads per kilo base million reads) of the selected gene was calculated based on the gff3 annotation file download from NCBI genome database (version: GRCh38.p2). Pearson correlation was applied for co-expression-network analysis based on the RPKM value of each gene from HOXC-AS3 knockdown group and control group (the same gene differ from this two group in the co-expression of related genes indicated that these two groups of genes are very important). For each pair of genes both in HOXC-AS3 knockdown and control group, we calculate the pearson correlation with p-value which was calculated and adjusted by Benjamini–Hochberg algorithm ([Benjamini Y, Hochberg Y. Controlling the false discovery rate: a practical and powerful approach to multiple testing[J]. Journal of the royal statistical society. Series B (Methodological), 1995: 289-300.]). Pearson correlation with FDR<0.05 was filtered to construct the co-expression-Network. Moreover, to study some properties of the networks, k-cores in graph theory were introduced as a method of simplifying graph topology analysis which could represent the core status of a RNA among phenotype related RNA group[^2^](#_ENREF_2). K-Core of the gene in HOXC-AS3 knockdown and control group was analyzed and different-K-core which indicated the core status between two groups was calculated. Gene with the largest K-Core difference was considered to be the core gene. Cytoscape (<http://www.cytoscape.org/>) was used to display the relationship of the Co-Expression-Network and core status of each gene.

1. Subramanian A, Tamayo P, Mootha VK, Mukherjee S, Ebert BL, Gillette MA, Paulovich A, Pomeroy SL, Golub TR, Lander ES, Mesirov JP. Gene set enrichment analysis: a knowledge-based approach for interpreting genome-wide expression profiles. Proc Natl Acad Sci U S A 2005;102:15545-50.

2. Ravasz E, Somera AL, Mongru DA, Oltvai ZN, Barabasi AL. Hierarchical organization of modularity in metabolic networks. Science 2002;297:1551-5.
